# Disaster Medicine Education for Medical Students: A Scoping Review

**DOI:** 10.7759/cureus.75035

**Published:** 2024-12-03

**Authors:** Haroon Rashid, Lykourgos Christos Alexakis, Irene Pereira

**Affiliations:** 1 Emergency Department, King's College Hospital NHS Foundation Trust, London, GBR; 2 Research, University of Piemonte Orientale, Novara, ITA; 3 Research, Research Group on Emergency and Disaster Medicine (ReGEDiM), Free University of Brussels, Brussels, BEL

**Keywords:** curriculum, disaster medicine, disaster medicine education, disaster planning, disasters, medical schools, medical students, undergraduate medical education

## Abstract

Education and training in disaster medicine for undergraduate medical students have been advocated for years in several countries. Despite the inclusion of disaster medicine into the medical curriculum being a reality in certain countries, such as the United States and Germany, it is still under scrutiny and yet to be embraced globally.

The objective of the study was to examine and map the range of literature and evidence available to support the inclusion of disaster education in the undergraduate medical curriculum and the identification of the related research gaps.

A scoping review was conducted using the Preferred Reporting Items for Systematic Reviews and Meta-Analyses (PRISMA) Extension for Scoping Reviews methodology proposed by the Joanna Briggs Institute. A study protocol was designed and distributed to all the authors. English articles published in peer-reviewed journals were searched across various databases for the period between 2004 and 2021. Abstracts and available full texts referring to the incorporation of disaster health educational programs into the undergraduate medical curriculum in different forms and formats were included.

Sixty-four articles were collected from 2004 to 2021. The bulk of the articles published was from the USA (n = 24), Germany (n = 6), Italy (n = 5), Saudi Arabia (n = 4), the UK (n = 3), India (n = 3), and Canada (n = 3). The types of articles included were mainly survey studies (n = 26) and pilot studies (n = 12). Forty-six (71.9%) articles included were based on courses taught on disaster medicine. The modes of teaching used were mainly face-to-face, simulation, and e-learning. The curriculum covered in the taught courses included general principles of disaster medicine (n = 34), chemical, biological, radiological, and nuclear/bioterrorism (n = 10), and pandemics (n = 2). Thirty-three articles were based on a single course while 13 articles studied more than one course. Further research in disaster health education, establishing a concise undergraduate medical curriculum globally, using technology and simulation, training the faculty, and developing interdisciplinary disaster education programs were the significant gaps identified in this study.

Available literature supports the inclusion of disaster medicine into the undergraduate medical curriculum globally. However, the boundaries and the inclusion criteria of the basic disaster health educational program into the existing undergraduate medical curriculum must be defined and agreed upon.

## Introduction and background

Disasters are adverse events that seriously disrupt the routine life of communities and exhaust local, regional, and national resources. Natural, man-made, and technological hazards can be the cause, and multiple injuries and casualties can happen. The available resources are insufficient to cope with the immediate need for medical care and other health consequences for the affected community at large. Current climate alterations, outbreak events, and complex emergencies, such as in war-affected regions, which are often multifaceted, constitute some of the other serious threats occurring [1,2].

Educating and training, community members in general, and health workers in particular, in disaster medicine is fundamental to disaster preparedness and planning [3-5].

The Sendai Framework for Disaster Risk Reduction (SFDRR) 2015-2030 considers the increase of disaster preparedness of the health systems and the training capacities in the field of disaster medicine as one of the priorities of the disaster risk reduction strategies [6].

Health professionals’ postgraduate programs in emergency and disaster health are still limited worldwide [5,7], but even more when considering this kind of program for medical students.

Education and training in disaster medicine for medical students have been advocated for years as an essential step of the educational program in several countries, especially after certain significant events or disasters occur, such as the February 27, 2010, earthquake in Chile and the March 11, 2011, Fukushima nuclear disaster in Japan [8-11]. Disaster medicine has already become a part of the formal curriculum at several international medical schools in Sweden, the United States, Germany, Italy, and Saudi Arabia [3,12-15].

However, the development of disaster health education for medical students is still under scrutiny, is heterogeneous, dispersed in topics, and with limitations such as defining measurable learning objectives, practical contents, learning strategies, shortfalls in the evaluation process, and other learning outcomes. Disaster education encompasses a broader body of knowledge not always considered enough when planning or organizing disaster health learning programs for the different levels of education and training [16-25].

As far as we know, Cummings et al. published the first systematic review on the subject in 2006 [26]. It included an analysis of the education of medical students in this area and the courses provided to physicians in the public and military sectors [26]. Two years earlier (2004), the same two first authors had already published a proposal for a disaster medicine curriculum for Canadian medical schools [27]. Disaster education, including both medical care and public health modules, provided to undergraduate medical students might give them more confidence, essential knowledge, and adequate basic skills, which could contribute to surging the local capacity when a disaster occurs [26,27].

A scoping review can provide an enlarged and comprehensive review and analysis of relevant studies on this theme published during the last 17 years [28].

Objectives

The objectives of the study are to examine and map the range of literature and evidence available to include disaster education in the curriculum of medical schools as well as to identify research gaps regarding the potential impacts of incorporating disaster education into undergraduate medical programs.

## Review

Methodology

Study Design

Frameworks for scoping reviews have been regularly developed and improved for almost 15 years by several authors to increase the clarity and quality of the review process [29,30]. The Preferred Reporting Items for Systematic Reviews and Meta-Analyses (PRISMA) Extension for Scoping Reviews proposed by the Joanna Briggs Institute was used to develop this study [30,31].

Searching for previous existence and eventual registration of a scoping review on this topic was made in the Open Science Framework. Registration for the final study will be made prospectively [32].

To ensure consistency, an a priori protocol was designed, discussed, and distributed to the student (main author) and co-authors before the data collection. Peer-reviewed search literature was independently compiled and a review of the data and extracted results was made. The third individual, serving as both a co-author and supervisor, was tasked with resolving significant inconsistencies or disparities and ensuring that all collected data adhered rigorously to the established research methodology. Guidance on search terms and the screening strategy was also sought from an expert librarian.

Eligibility/Inclusion Criteria

The articles included in the study were published in English in peer-reviewed journals, between January 2004 and December 2021, with abstracts and full text available, and with a focus on any stage/phase of the pre-graduated physician education level (basic or advanced years of learning), and regional, local, or international context. Relevant reference lists and hand-searched articles were also included for the same period.

*Population, Concept, and Context (PCC* *) Approach*

A PCC approach was used as proposed by the Joanna Briggs Institute [30]: population (undergraduate medical students, any stage/phase of medical education), concept (medical curriculum or learning programs in disaster health education, disaster medicine, and/or disaster planning or disaster preparedness, mentions to evaluation or effectiveness of programs or curricula tested or applied in the study), and context (any setting where undergraduate medical students were part of the target population, regional, local, or international context) were considered in the inclusion criteria.

Exclusion Criteria

Commentaries, editorials, book chapters, dissertation thesis, and conference abstracts or proceedings were also excluded from the study. Similarly, other grey literature was not included. Other health area students, such as nursing, pharmaceutical, paramedic, or other than physicians, were excluded.

Data Sources

Only peer-reviewed articles published from January 2004 to December 2021 were included. The reason for this specific period was that the first main systematic review on the topic was conducted in 2006 (medical students were a part of the studied population), and the first specific paper on the issue from the same authors was published in 2004 [26,27]. The data were collected from the electronic literature databases, including PubMed/MEDLINE (National Center for Biotechnology Information, National Institutes of Health, Bethesda, Maryland, USA), Scopus (Elsevier, Amsterdam, Netherlands), Web of Science Core Collection (Clarivate Analytics, Philadelphia, Pennsylvania, USA), and from the search engine Google Scholar (Google Inc., Mountain View, California, USA). Other sources included relevant reference lists from other papers and hand-searched articles from the journal “International Journal of Disaster Medicine” (Taylor & Francis).

Search Words and Phrases

The keywords searched during screening and article search contemplated terms such as “medical student(s)”, “undergraduate medical students”, “medical school(s)”, “curriculum”, disaster(s)”, “disaster medicine”, “disaster planning”, “disaster preparedness”, “disaster health education”, and “disaster medicine education” (Appendix 1).

Search and Screening Strategy

The search was conducted according to the current international research recommendations for each selected database (Appendix 2). The terms searched included the most relevant MeSH terms, relevant keywords, and free terms related to the type of study design. The Boolean operators were used to combine or exclude the search terms. The articles included in the study were screened in two phases. In the first phase, the articles were screened based on the relevance of the title and abstract, and then in the second one, full-text screening was performed.

Data Extraction

Mendeley (Elsevier B.V., Amsterdam, Netherlands) was used to compile the references, and Microsoft Excel 2007, version 12.0.4518.1014 (Microsoft Corporation, Redmond, Washington, USA) spreadsheet was used for data extraction.

Ethical Approval

Ethical approval is not a requisite for this kind of study. This study was conducted as a research thesis, a part of the Advanced Masters of Science in Disaster Medicine (European Masters in Disaster Medicine), and was approved by the Strategic Management Board of the Program.

Results

**Figure 1:**
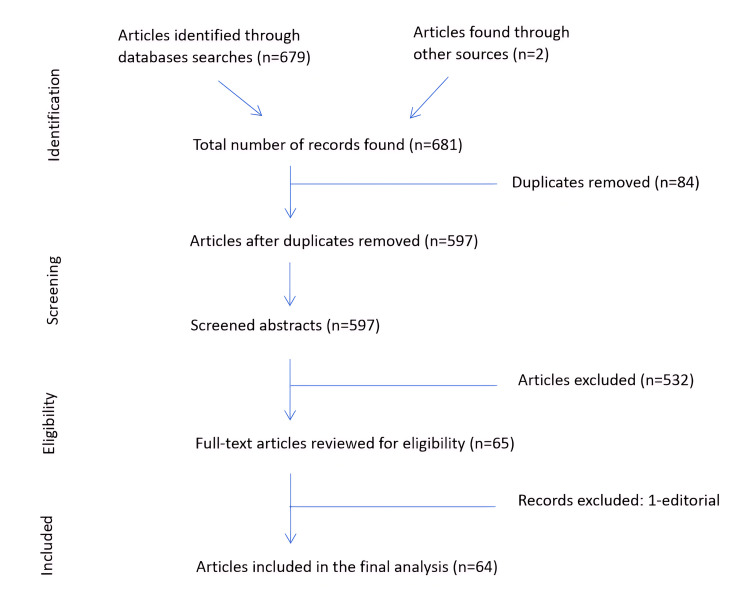
PRISMA flow chart for the selected studies. PRISMA: Preferred Reporting Items for Systematic Reviews and Meta-Analyses.

Based on the inclusion criteria, a total of 681 articles were identified during the search process in the first phase (Figure 1). Two articles were manually screened from the International Journal of Disaster Medicine. Sixty-four published study articles were finally selected based on the selection criteria. Studies' authors, year of publication, country of origin (where the study was published or conducted), type of studies, study population, adopted study method, and study analysis method used are presented in Table 1. The selected articles were reviewed on whether they supported the inclusion of disaster medicine in the undergraduate medical curriculum and for any study gaps identified by those studies.

**Table 1:** Summary of the selected study articles (n = 64). AAMC: Association of American Medical Colleges; MCQ: multiple-choice questions; OSCE: Objective Structured Clinical Examination.

Study reference	Year	Country	Type of study	Study population	Study method used	Study analysis method
Cummings G et al. [27]	2004	Canada	Survey	Medical students (undergraduate, fellowship, postgraduate)	Survey questionnaire	Survey analysis
Parrish AR et al. [33]	2005	USA	Interventional study	Medical students (72 2nd year - 2003-2004; 2004-2005) and course faculty (6 military experts)	Descriptive review /post-test and post-attitudinal questionnaire	Descriptive analysis/self-evaluation, attitudes & knowledge of the preparedness
Cummings GE et al. [34]	2005	Canada	Survey	10 undergraduate and 14 fellowship program representatives (48 representatives and 15 Canadian medical schools)	Survey questionnaire (internet-based)	Survey analysis
Markenson D et al. [35]	2005	USA	Review study	4 medical schools (physicians, public health, dental, nursing)	Author review of existing practices to deal with bioterrorism and development of core competencies for future	Expert analysis
Cummings G et al. [26]	2006	Canada	Systematic review	Medical students, physicians, medical military	Research studies critical review	Descriptive analysis
Subbarao I et al. [25]	2008	USA	Systematic review and Delphi study	All health professionals including medical students	Research studies expert working group review (18 persons)/3 round Delphi study	Review process + 3-stage Delphi process
Altintas KH et al. [18]	2009	Turkey	Survey	Medical students (191 1st-year and 232 6th-year students)	Survey questionnaire	Chi-square test
Kaiser HE et al. [36]	2009	USA	Survey	523 medical students	Web-based survey questionnaire	Descriptive data analysis
Merlin MA et al. [37]	2010	USA	Survey	49 (4th year) medical students	Pre- and post-rotation questionnaires	Simple paired t-test
Kaji AH et al. [38]	2010	USA	Pilot study	6 medical students (4th year)	Summative feedback questionnaire-structure & utility of the course, 73-item oral exam, final lecture present	Survey analysis
Pfenninger EG et al. [39]	2010	Germany	Pilot study	Medical students, course faculty, and representatives of professional, societal, and government bodies	Curriculum development 6-step approach. Pilot testing (faculty & student feedback pilot versions of curriculum) during 2 years (pilot courses). Survey of all physicians (n = 477), German county disaster management agencies & district health authorities for additional input into disaster medicine needs competencies. Pre- and post-course questionnaire (oral &/or written final examination, comparing test results pre & post program, student & faculty summative and formative course evaluation	Survey analysis
Franc-Law J et al. [40]	2010	Italy	Prospective observational cohort	22 medical students by year of education (random 50% interventional group; 50% control group)	Performance indicator scoring system, student evaluation questionnaire	R statistical analysis, χ2 test
Kaji AH et al. [41]	2010	USA	Descriptive and qualitative analysis	Medical students	Scored evaluation of the seminar, focus group discussion	Descriptive and qualitative analysis
Scott LA et al. [42]	2010	USA	Pilot study-Delphi study	Medical students (4th year)	Pre- and post-course questionnaire	Modified Delphi technique
Saiboon IM et al. [43]	2011	Malaysia	Survey	45 medical students (5th year)	Pre- and post-lecture questionnaire (14 points)	Paired t-test
Carney JK et al. [44]	2011	USA	Survey	3 classes of 115 medical students each/different institutions/different years: 1st, 2nd, and 4th year	Survey of workshops	Survey analysis
Datta R et al. [45]	2012	India	Review article	Undergraduate and postgraduate medical students and doctors	Author review, pre and post-lecture questionnaire	Author opinion, survey analysis
Smith J et al. [46]	2012	USA	Pilot survey	Educational liaison of 29 medical schools accredited by AAMC	Electronic on-line survey	Survey analysis
Scott LA et al. [47]	2012	USA	Delphi Study	10 medical students and 17 emergency physicians, nurses, and emergency managers	Task force-Delphi process (development of competencies for the course) + small group exercises	Task force-Delphi process (development of competencies for the course) + small group exercises
Markenson D et al. [48]	2013	USA	Survey	Undergraduate medical, dental, and nursing students	Web-administered survey questionnaire	Survey analysis/comparative study
Su T et al. [49]	2013	China	Survey	547 health professionals, 456 medical students, 1526 local residents	Self-reporting questionnaire survey	Descriptive analysis, chi-square, linear regression analysis
Ragazzoni L et al. [50]	2013	Italy	Survey	Medical students registered as members of the International Federation of Medical Students' Associations (undergraduate 1st to 6th year) and supplementary year (13) and post-graduate (4)	Web-based survey questionnaire (14 questions/4 groups)	Survey analysis
Jasper E et al. [51]	2013	USA	Survey	130 new incoming interns at an academic teaching hospital	Questionnaires	Survey analysis
Grossi F et al. [52]	2014	Italy	Case-control study	Medical students = 125 (3rd to 6th year)/2 groups - 125 experimental group and 105 control (grade 44 students)	Case/control study (2 groups), pre and post-knowledge and attitude tests post a peer-reviewed course	Descriptive analysis, t-test, Wilcoxon–Mann–Whitney, and Wilcoxon signed rank test
Ingrassia PL et al. [14]	2014	Italy	Pilot study	524 - 4th, 5th, and 6th-year medical students (21 medical schools corresponding to 52.5% of all Italian med schools)	Development course (six-step approach) and satisfaction and knowledge questionnaires + pre-test + post-test after completing the course (25 MCQ)	Survey analysis
Afzali M et al. [53]	2015	Denmark	Descriptive analysis	66 medical students (3 groups of 16 and 1 group of 18)	Post-course student evaluation of the course and self-assessment	Descriptive analysis
Harmer A et al. [54]	2015	UK	Descriptive, case study	All director’s global health programs directors, 15 universities, UK undergraduate (6 programs) and postgraduate global health students (25 programs, both medical and non-medical)	Survey questionnaire	Survey analysis
Pollard KA et al. [55]	2015	USA	Pilot study	52 medical students + pre-medical students, resident doctors (total = 45)	Student performance evaluation, pre and post-test questionnaire	Paired t-test
Mortelmans LJ et al. [56]	2015	Netherlands	Survey	4408 (university total) - 5th and 6th senior medical students (from 6 to 8 faculties)	Descriptive cross-sectional survey (10 theoretical and practical case questions (validated))	Survey analysis
Bajow N et al. [57]	2015	Saudi Arabia	Pilot study	Health expert stakeholders and international expert communities on disaster medicine, medical undergraduates	Questionnaire and interviews	Survey analysis
Bajow N et al. [58]	2015	Saudi Arabia	Survey	30 medical schools - academic medical affairs directors	Online survey questionnaire (25 items)	Descriptive analysis, t-test, chi-square
Mohamed-Ahmed R et al. [59]	2015	UK	Interventional study	27 students	Pre and post-test sessions evaluation forms (self-rate confidence in 8 learning domains)/qualitative + quantitative	Descriptive quantitative analysis, Wilcoxon signed-rank test
Bajow N et al. [15]	2016	Saudi Arabia	Pilot study	29 medical students (15 male; 14 female) of 4th, 5th, and 6th year at Jazan University, Saudi Arabia	Develop a 5-stage approach (video lecture, workshops, group discussion; role-playing, mock & experimental learning, computer games, training for community education). Pre and post-course questionnaire, self-assessment, 3rd level of Kirkpatrick's evaluation	Wilcoxon test for paired samples
Barrimah I et al. [60]	2016	Saudi Arabia	Survey	250 medical students (clinical phase) and teachers of the college, emergency physicians, and health administrators (interviews)	Quantitative and qualitative analysis of questionnaire + interviews	Survey analysis and SPSS for qualitative analysis
Mortelmans LJ et al. [61]	2016	Belgium	Survey and comparative study	All military students in medical sciences (not only medicine/27% senior level) & 999 civilian medical students	10 theoretical and practical questions + comparison with previous similar data from med students	Survey comparative analysis + statistical analysis
Cole LA et al. [62]	2016	USA	Survey	14 medical students (4th year)	Post-course student evaluation of the course	Descriptive analysis
Wunderlich R et al. [13]	2017	Germany	Prospective, cross-sectional, observational survey	All medical students of 37 German medical schools	Web-based, purpose-designed questionnaire	Survey analysis statistical analysis (descriptive + quantitative)
Jasper EH et al. [63]	2017	USA	Survey	503 medical students (1st year)	Questionnaire (23 questions)	Survey analysis
Prihatiningsih TS et al. [64]	2017	Indonesia	Randomized controlled trial	72 (3rd year) students of medicine, nursing, and health and nutrition programs at 1 university in Indonesia, 36 randomized interventional studies, and 36 randomized for control group	Randomized controlled trial	Descriptive mixed-method analysis
Yasui K et al. [11]	2017	Japan	Review article	Undergraduate and postgraduate medical students	Narrative review of published articles in several databases and focus on 3 new representatives of the curriculum of Japanese medical education that started after the Great East Japan Earthquake	Author analysis
Kim TE et al. [65]	2017	USA	Interventional study	402 undergraduate medical, nursing, and pharmacy students	Pre-course and post-course assessments online (performance, leadership, teamwork, course satisfaction)	Qualitative analysis
Patel R et al. [66]	2017	USA	Survey	631 medical, nursing, and pharmacy students	Survey questionnaire	Two-way ANOVA, post hoc analysis
Verson J et al. [67]	2018	USA	Pilot study	28 medical students (1st year)	Exercise via email city-wide drill simulating a large-scale aerosolized release of Bacillus anthracis/arriving ED hospital post-call	Author opinion, descriptive analysis of drill
Drees S et al. [68]	2018	Germany	Descriptive study	89 Medical students	Author review of 7 workshops and the game "AFTERSHOCK" + survey "student self-evaluation" + written evaluation	Survey analysis
Wiesner L et al. [21]	2018	USA	Interventional study	2 cohort of studies - 30 medical students, 1st cohort and 51 medical students, 2nd cohort (from all class years) (2nd-year medical students)	10 questions of multiple-choice (prior to and after each training session) + statistical analysis	Paired t-test
Patel VM et al. [69]	2018	USA	Interventional study/survey	55 medical students	Pre-elective, post-lesson, and post-elective questionnaire	Survey analysis
Scott LA et al. [70]	2018	USA	Interventional study/survey	708 participants (31.9% medical students, 49.9% physicians, 7.2% nurses, and 11% allied health professionals) enrolled in a university between 2011 and 2014	Facilitator observation, pre and post-testing, and a course evaluation	Descriptive analysis, unpaired t-tests
Kommor MB et al. [12]	2019	USA	Pilot study	68 medical students (2nd year - 47; third year - 21)	Retrospective questionnaire (survey for evaluation of the program)	Survey analysis
Back DA et al. [71]	2019	German	Pilot study	51 medical students (3rd year)	Pre and post-course multiple-choice tests and questionnaire	Survey analysis
Rezaee R et al. [72]	2019	Iran	Delphi survey	15 medical and disaster experts	Delphi questionnaire (2 rounds)	Survey analysis
Gouda P et al. [73]	2020	Ireland	Survey	About 830 medical students of an Ireland university	Online survey questionnaire	Pearson’s chi-square correlation, independent t-tests, and multinomial regression
Ragazzoni L et al. [74]	2020	Italy	Interventional study	2316 medical students from 2014 to 2018/41 medical students become trainers (2013-2018) and students’ teacher	Demographic questionnaires, pre-test and post-tests, and satisfaction questionnaires	Survey analysis
Tsai YD et al. [75]	2020	Taiwan	Pilot study	230 medical students	Questionnaire (knowledge at the beginning and end of training) - 10 MCQs	Survey analysis
Gillani AH et al. [76]	2020	Pakistan	Survey	310 students, medical and pharmacy undergraduate students	Pretested and validated self-administered questionnaire	Independent t-test, one-way ANOVA, Pearson correlation, and regression analyses
Panda M et al. [77]	2020	India	Survey	Medical students	Predesigned semi-structured questionnaire	Survey analysis
Rajesh G et al. [78]	2020	India	Survey	437 final-year students pursuing medicine (114), dentistry (86), nursing (42), physiotherapy (53), pharmacy (58), Ayurveda (23), and homeopathy (61) of 7 institutions in Mangalore and health professions	Questionnaire - 42 items (26 = knowledge; 8 = attitude; 8 = behavior)	Descriptive analysis, linear regression analysis
Ponampalam R et al. [79]	2021	Singapore	Survey	250 medical students/10 in rotation every 3 months	MCQs, quizzes, and OSCE	Survey analysis
Ashcroft J et al. [80]	2021	UK	Systematic review	23 studies met the inclusion criteria	Research studies critical review	Descriptive analysis
Al-Ziftawi NH et al. [81]	2021	Qatar	Survey	Medical, pharmacy, and health sciences students	Pretested and pre-validated survey questionnaire	Student's t-test, analysis of variance, correlation, and linear regression
Hermann S et al. [82]	2021	Germany	Prospective and cross-sectional survey	102 medical students (5 courses from 2018-2020)/(3rd, 4th, and 5th year)	3 survey tools	Paired sample t-test
Park H et al. [83]	2021	S. Korea	Scoping review	57 papers met inclusion criteria	Arksey and O’Malley’s protocol methodology	Quantitative and thematic content analysis
Kasselmann N et al. [84]	2021	Germany	Survey	Deans of 36 German medical schools	Online questionnaire	Survey analysis, descriptive statistics
Saiboon IM et al. [85]	2021	Malaysia	Prospective cross-sectional survey	168 pre-clinical year medical students	Validated online questionnaire	Survey analysis
Gable BD et al. [86]	2021	USA	Survey	Medical students	Pre and post-test questionnaire	Paired analysis, Wilcoxon signed-rank sum test

**Figure 2:**
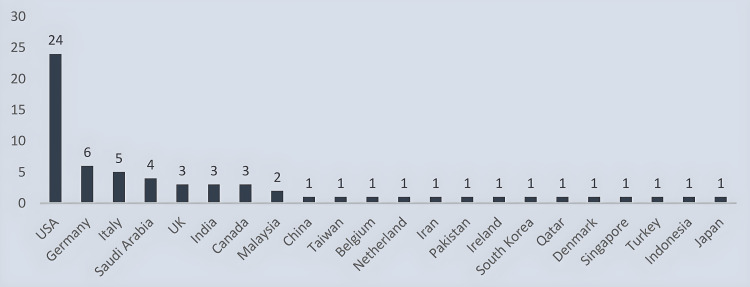
Country of origin of the selected studies (n = 64).

After passing through all the screening phases, 64 articles were selected to be included in this scoping review. Most of the articles were published in the USA (n = 24/38%). Six articles were from Germany while four articles each were of Italian and Saudi Arabian origin. There were three articles each from the UK, India, and Canada. Malaysia contributed two articles while one article each from China, Taiwan, Belgium, Netherlands, Iran, Pakistan, Ireland, South Korea, Qatar, Singapore, Denmark, Turkey, Indonesia, and Japan was made part of the study (Figure 2).

**Figure 3:**
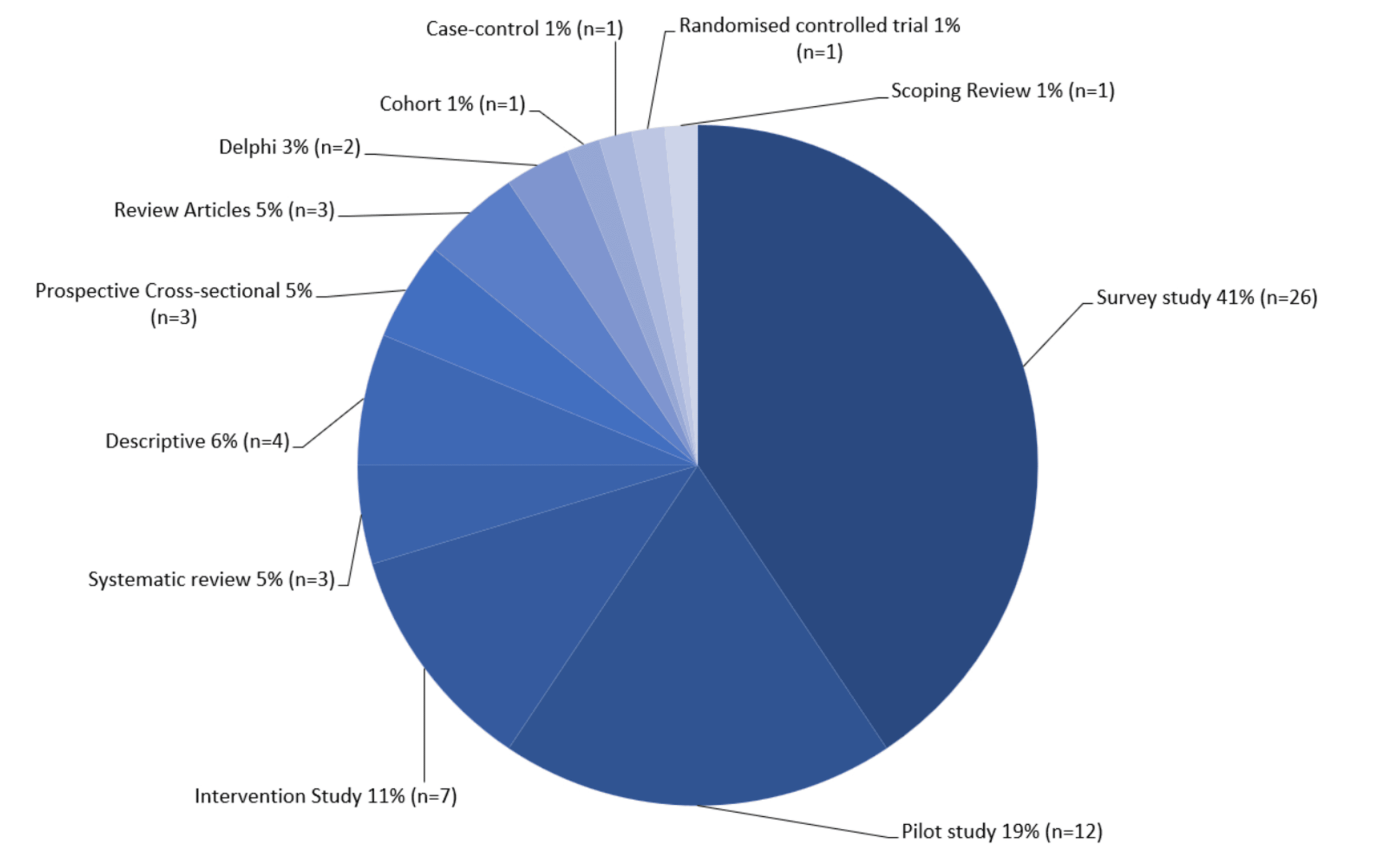
Types of selected studies (n = 64).

Twenty-six (41%) of the studies included were survey studies while 12 (19%) were pilot studies. Seven interventional studies, four descriptive studies, and three systematic reviews were included in this scoping review. In addition, three were cross-sectional studies and review articles. Two Delphi studies, one scoping review, one case-control study, one cohort study, and one randomized control study were also made part of this scoping review (Figure 3).

**Figure 4:**
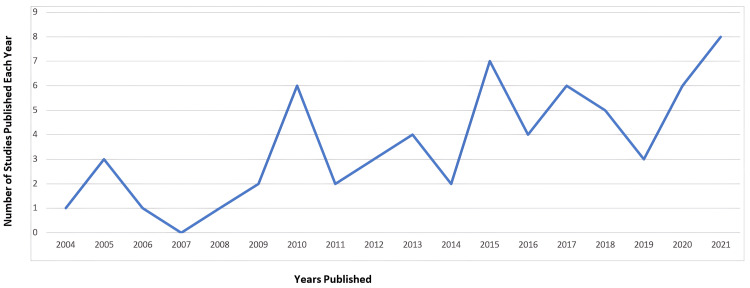
Year of publication of the selected studies (n = 64).

A directly proportional relation was noted between the number of articles published on disaster medical education for undergraduate medical students and every passing year. The highest number of articles included in the study were published in the year 2021 (n = 8) (Figure 4).

Overall, two main groups of articles were identified: one focusing on the organization, implementation, and follow-up of courses in disaster medicine for medical students (n = 46/71.9%), and the other primarily addressing self-perception or self-knowledge of students/other health professionals student respondents.

Mode of Education for the Courses in the Selected Studies

There were 46 articles in this scoping review involving educational courses on disaster medicine using different teaching modalities. Most of the study courses were a combination of face-to-face lectures and simulation (n = 31) whereas other taught courses used e-learning in addition to face-to-face and simulation methods (n = 8). The courses in two studies were based only on e-learning while in one study, the course was taught using simulation methods only. Three studies used a face-to-face and e-learning blended approach to teach their students while in one study, the course was taught via an e-learning and simulation method.

Curriculum of the Taught Courses in the Studies Selected

The curriculum taught in these courses was mainly general principles of disaster medicine (n = 34) [87] while chemical, biological, radiological, and nuclear disasters or bioterrorism was also a favorite topic taught in these courses (n = 10). Two articles included in this review were focused on COVID-19 and pandemics, i.e., health disasters.

Mandatory or Optional and Single or Multiple Taught Courses in the Selected Studies

Out of 46 articles based on taught courses, 36 articles studied optional or non-compulsory courses, i.e., not part of the mandatory curriculum, eight articles included courses based on mandatory curriculum while two articles had a combination of both mandatory and optional courses. Thirty-three articles were based on a single course while 13 articles made more than one course part of their study.

All the study articles suggested the inclusion of disaster medicine into the undergraduate medical curriculum.

Discussion

Education of disaster medicine for undergraduate medical students has been well advocated [13-15]. Education in disaster medicine has grown in popularity over recent times.

Disaster medicine education for undergraduate medical students is a relatively new and evolving field. Many studies included in this article recommend further research in disaster medicine education at the undergraduate level. Cummings et al. (2006) proposed that the available disaster medicine courses were well-documented and needed to be further studied, peer-reviewed, and indexed [26]. Jasper et al. (2017) recommended that the focus of future education research should be on developing interdisciplinary curricula to ensure that disaster medicine is taught across all undergraduate years [63]. Further research with a large sample size was suggested by Grossi et al. (2014) to differentiate between the traditional vertical teaching method and peer-led training in disaster medicine education [52]. Kim et al. (2017) suggested continuous research to demonstrate sustained improvement in the course and its clinical benefits [65]. Merlin et al. (2010) and Scott et al. (2018) emphasized the importance and need to find the areas of further research on disaster medicine education [37,70], while Gouda et al. (2020) pointed out the need for research and further experiences to establish disaster training programs and to find out appropriate roles of medical students during a disaster [73]. Scott et al. (2010) advocated the necessity of research to validate core competencies and to set out performance-based education goals in disaster medicine [42].

A blended approach, combining traditional lecture and simulation techniques, has been suggested by Ingrassia et al., Drees et al., and Panda et al. for better learning outcomes [14,68,77]. Similarly, Herman et al. (2021) studied a broader scope of virtual reality training in disaster medicine [82]. Park et al. (2021) highlighted the use of telemedicine, which helped a great deal during the COVID-19 pandemic [83]. Saiboon et al. (2011), Datta et al. (2012), Su et al. (2013), Patel et al. (2018), and Kasselmann et al. (2021) supported the use of e-learning and simulation for disaster medical training for ease of use and efficacy [43,45,49,69,84]. Saiboon et al. (2021) suggested that e-learning was beneficial in cognitive learning, but the complex decision-making knowledge was better taught as practical face-to-face learning [85].

Ragazzoni et al. (2013), Jasper et al. (2013), Mortelmans et al. (2015), Barrimah et al. (2016), Gouda et al. (2019), Gillani et al. (2020), and Al-Ziftawi et al. (2021) found that the medical undergraduates expressed keen interest in learning disaster medicine and willingness to be a part of the disaster response team in case of an adverse event [50,51,56,60,73,76,81]. Moreover, Gillani et al. (2020) showed a positive correlation between knowledge and attitude toward disaster medicine learning among healthcare professionals [76].

Bajow et al. (2015) suggested that most of the participating medical schools were willing to make disaster education a part of the undergraduate curriculum but were unable to do so due to the lack of trained staff [57]. Prihatiningsih et al. (2017) cited that already saturated undergraduate curricula, lack of financial resources, and assistance from the faculty and administration were the hurdles to implementing inter-professional disaster education [64].

Two articles studied military and civilian disaster training. Mortelmans et al. (2016) compared military with civilian medical undergraduates and found that military undergraduates had an overall better grasp and were more competent in dealing with a disaster as compared to civilian undergraduates [56] while Back et al. (2019) suggested that merging civilian and military disaster training programs would bring forth collaborated and multifaceted approach and might open up new avenues in disaster education [71].

Many educational and training courses, workshops, and seminars have been run in different countries, specially designed for undergraduate medical students [11,12,21]. Despite the literature evidence supporting the inclusion of disaster medical education in the undergraduate medical curriculum, disaster medicine has not yet been accepted worldwide by numerous medical schools, with some exceptions in the USA, Germany, Italy, and Saudi Arabia [13,14,21,39,50,58,84]. The Association of American Medical Colleges has strongly recommended including disaster medicine in the undergraduate medical curriculum to prepare health professionals for mass casualty incidence response [12,84]. The main reasons for disaster medical education not being part of the undergraduate medical curriculum were lack of resources and absence of organizational structure [84], scarcity of trained staff to train the medical students [57,58], lack of awareness about disaster medicine [50,60], and no formulated curriculum framework for the undergraduate medical students [14,51].

We still noticed that most of the studies do not have a strong evidence-based hierarchy. No study aimed at evaluating the validity of the course except one Italian study in 2014 by Ingrassia et al. (2014) that was conducted to evaluate the validity of disaster medicine training for undergraduate medical students in Italy [14]. It highlights the absence of a valid and uniformly accredited disaster medicine curriculum for medical undergraduates globally. The articles based on taught courses included in this scoping review had their own limitations. The barriers or limitations described in these articles were mainly selection bias, participant bias, time constraints, and a small study population affecting the validation of the study.

Limitations

This study did not include articles in languages other than English. Another important limitation is that grey literature was not included in the literature search. Both could have eventually limited the number of additional information important to the study. We believe that most cited and referenced papers were mentioned, and one cannot neglect that used search strategies could influence the number of results.

## Conclusions

Disaster medicine education has not yet been embraced as a part of the undergraduate curriculum. If this subject is introduced gradually at different stages of medical training and on a global scale, it could have a huge impact.

This scoping review identified the need for (a) more research in the field of disaster medicine education, (b) better-designed quality studies with bigger samples, (c) planning interdisciplinary and competency-based curricula for disaster medicine training, and (d) use of the modern technology and simulations for training.

## Appendices

Appendix 1

**Table 2:** Population, concept, and context (PCC) approach construction.

Population	Concept	Context
MESH terms	MESH terms	MESH terms
Student*/medical	Disaster*	School*/medical
Undergraduate medical student*	Disaster medicine	Education/medical
	Disaster planning	
Keywords	Keywords	Keywords
Undergraduate	Disaster preparedness	Medical schools
Medical students	Curricul*	
Phrases	Phrases	Phrases
	Disaster medicine education	
	Disaster health education	
	Disaster curricula	

Appendix 2

**Table 3:** Search strategy.

PubMed/MEDLINE	Scopus	Web of Science Core Collection	Google Scholar
A first basic search with disaster medicine curriculum and medical students. Filters: Abstract, Full text, English, from 2004/1/1 - 2021/12/31. Second search (((disaster medicine OR “disaster planning” OR “disaster preparedness” OR (disaster medicine OR disaster* OR disaster planning [MeSH Terms])) AND “disaster medicine education” OR “disaster curricula”)) AND “(“medical students” OR undergraduate). Filters: Abstract, Free Full Text, from 2004/1/1 - 2021/12/31	(TITLE-ABS-KEY {disaster*) OR TITLE-ABS-KEY ({disaster medicine} OR {disaster planning} OR {disaster preparedness}) OR TITLE-ABS-KEY ({disaster health education} OR {disaster medical education} OR {disaster curricul*}) AND TITLE-ABS-KEY ("undergraduate*" OR {undergraduate medical students} OR "medical student*") AND TITLE-ABS-KEY ("medical school" OR "medical university")) AND PUBYEAR > 2003 AND PUBYEAR <2022 AND (LIMIT-TO (SUBJAREA, "MEDI")) AND ( LIMIT-TO ( PUBSTAGE, "final")) AND (LIMIT-TO (LANGUAGE, "English))	TS= disaster* OR “disaster medicine” OR “disaster planning” OR “disaster preparedness” AND TS = ”disaster health education” OR disaster medicine education” OR “curricul*” AND TS = ”medical student” OR “undergraduate” AND TS = ”medical school” OR “medical faculty”. From 2004-01-01 to 2021-12-31 English	Advanced search: Find articles: - with all the words: "disaster medical education" OR "disaster health education" OR "disaster preparedness" AND "medical students" AND " "medical schools" - with the exact phrase: "disaster health education" - with at least one of the words: education “disaster medicine” - not: Nurses. Anywhere in the text with citations 2004-2021, English language
